# Signatures of Selection in the Genomes of Commercial and Non-Commercial Chicken Breeds

**DOI:** 10.1371/journal.pone.0032720

**Published:** 2012-02-27

**Authors:** Martin G. Elferink, Hendrik-Jan Megens, Addie Vereijken, Xiaoxiang Hu, Richard P. M. A. Crooijmans, Martien A. M. Groenen

**Affiliations:** 1 Animal Breeding and Genomics Centre, Wageningen University and Research Centre, Wageningen, The Netherlands; 2 Breeding Research and Technology Centre, Hendrix Genetics, Boxmeer, The Netherlands; 3 State Key Laboratory for Agrobiotechnology, China Agricultural University, Beijing, China; Massachusetts General Hospital, United States of America

## Abstract

Identifying genomics regions that are affected by selection is important to understand the domestication and selection history of the domesticated chicken, as well as understanding molecular pathways underlying phenotypic traits and breeding goals. While whole-genome approaches, either high-density SNP chips or massively parallel sequencing, have been successfully applied to identify evidence for selective sweeps in chicken, it has been difficult to distinguish patterns of selection and stochastic and breed specific effects. Here we present a study to identify selective sweeps in a large number of chicken breeds (67 in total) using a high-density (58 K) SNP chip. We analyzed commercial chickens representing all major breeding goals. In addition, we analyzed non-commercial chicken diversity for almost all recognized traditional Dutch breeds and a selection of representative breeds from China. Based on their shared history or breeding goal we *in silico* grouped the breeds into 14 breed groups. We identified 396 chromosomal regions that show suggestive evidence of selection in at least one breed group with 26 of these regions showing strong evidence of selection. Of these 26 regions, 13 were previously described and 13 yield new candidate genes for performance traits in chicken. Our approach demonstrates the strength of including many different populations with similar, and breed groups with different selection histories to reduce stochastic effects based on single populations.

## Introduction

Domesticated chicken breeds are diverse with differences in morphology, physiology and behavior [1]. Traditional breeds are mostly kept for ornamental purposes and display a large diversity in morphological phenotypes between breeds. Charles Darwin already noticed the large diversity of phenotypes within the chicken and assumed a single-origin for the domesticated chicken descending from *Gallus gallus* (Red Junglefowl (RJF)) [2]. Although a single-origin was supported by many studies (e.g. [3]–[6]), it was debated by others [7], [8]. Archeological findings suggest that multiple domestication events and multiple geographical regions were involved in the establishment of the domesticated chicken [9] which is supported by molecular genetic evidence [10]–[11]. Moreover, molecular evidence supports genetic contributions from other Junglefowl species to current domesticated chickens. For instance, the yellow skin locus present in several domestic chicken breeds most likely originated from *Gallus sonneratii* (Grey Junglefowl) [12].

The chicken may have initially been domesticated for cultural reasons such as religion, decoration, and cock fighting instead of a food resource [13]. Despite selective breeding that has been documented as early as Roman times [13], it was not until the 20^th^ century that commercial breeding companies selected strongly for production traits. Specialized breeding lines were intensely selected for either growth traits (meat production) or reproductive traits (egg-laying) which led to a massive selection response to those breeding goals [13]–[15]. The vast majority of commercial chicken breeds in Europe and Northern America are established from only a handful of breeds. Although non-commercial breeds are still present, effective population sizes are generally small (tens to hundreds [6]) and many breeds are threatened with inbreeding or extinction which will result in a decreased biodiversity in chicken [16].

The domestication of the chicken created population bottlenecks and subsequent population growth, admixture of populations, inbreeding, genetic drift, and selective breeding. As a consequence of these demographic and selective events the genetic variation within the domesticated chicken genome must have changed from its ancestral state. Selection on desirable alleles will lead to a reduction or loss in nucleotide diversity at and near the selected locus, often referred to as hitch-hiking or selective sweep [17], [18]. Selective breeding in commercial breeds has increased production but has also reduced resistance to infectious disease [19] and increased skeletal deformities [20], osteoporosis [21], and the pulmonary hypertension syndrome [22]–[25]. These undesirable traits and diseases may be the result of negative pleiotropic effects of the alleles under selection or from genetic hitch-hiking of undesirable alleles with the alleles under selection. To understand these hitch-hiking effects on genetic diversity and negative pleiotropy it is essential to identify regions and genes that have been under selection. Furthermore, this information should aid in understanding the domestication and selection history of the domesticated chicken and how molecular pathways may have altered compared to the ancestral state, thereby facilitating the discovery of important genes and further improvement of production traits.

A recent study that applied a massively parallel sequencing strategy identified chromosomal regions and genes putatively under selection during chicken domestication and selective breeding [1]. However, this study only focused on a small number of breeds, making generalizations on selection history throughout the domesticated and wild chickens uncertain.

In contrast, we aimed to make a broad assessment of the effects of selection histories in domesticated chicken. Therefore we analyzed commercial chickens representing all major breeding goals. In addition, we analyzed non-commercial chicken diversity for most traditional Dutch breeds and a selection of representative breeds from China. In addition, several non-domesticated chicken populations were analyzed as well as a related non-domesticated species (*Gallus lafayetii*). This sample of 67 commercial and non-commercial breeds was assessed for signatures of selection in the genome using information of 57,628 SNPs genotyped on pooled DNA samples. Using multiple populations for each breed will decrease the influence of stochastic effects such as genetic drift that may result from using just a single population. Furthermore, this strategy may reveal larger scale breed or breeding goal specific selection histories, rather than population-specific selection histories, potentially making it easier to interpret signatures of selection.

## Materials and Methods

### Data collection

The 67 breeds represent multiple populations of commercial broiler dam (n = 5) and sire (n = 8) lines, commercial white (n = 11) and brown (n = 11) egg-layers, Dutch traditional breeds (n = 19), and Chinese breeds (n = 10) (Table 1). Two subspecies from *Gallus gallus* (*Gallus gallus gallus, Gallus gallus spadiceus*) were also included whilst the *Gallus lafayetii* was used as an outgroup (Table 1). Individual samples were collected from the breeds varying from 8 to 75 individuals per breed (Table 1). Pools were made by either adding equal amounts of blood before DNA extraction or by adding equal amounts of DNA for each individual within each breed. DNA concentrations were measured by a NanoDrop spectrophotometer. Blood collection for the commercial breeds was carried out by licensed and authorized personnel under approval of Hendrix Genetics (Boxmeer, the Netherlands). For the Dutch traditional breeds, *Gallus gallus gallus, Gallus gallus spadiceus*, and *Gallus lafayetii* DNA samples were used from previous studies [6], [26], [27]. The raw genotype data for the Chinese breeds were provided by the State Key Laboratory for Agrobiotechnology, China Agricultural University, Beijing, China free of charge. Although the genotype experiments were performed for the purpose of our study, the DNA samples were not collected for the purpose of our study. The DNA samples were taken for other purposes at the China Agricultural University.

**Table 1:** Information on the genotyped breeds.

	Breed name	# ind^1^	Hp^2^	Origin^3^	Breed groups^4^			
**Junglefowls**	*G. lafayetii*	11	0.04	Sri Lanka	Outgroup			
	*G. g. gallus* 5	30	0.37	Thailand	NDM			
	*G. g. spadiceus* 5	30	0.36	Thailand	NDM			
**Broiler**	Broiler sire 1	75	0.42	commercial	DM	CM	BR	BRS
**sire line**	Broiler sire 2	75	0.43	commercial	DM	CM	BR	BRS
	Broiler sire 3	75	0.43	commercial	DM	CM	BR	BRS
	Broiler sire 4	75	0.42	commercial	DM	CM	BR	BRS
	Broiler sire 5	75	0.39	commercial	DM	CM	BR	BRS
	Broiler sire 6	75	0.41	commercial	DM	CM	BR	BRS
	Broiler sire 7	75	0.42	commercial	DM	CM	BR	BRS
	Broiler sire 8	48	0.39	commercial	DM	CM	BR	BRS
**Broiler**	Broiler dam 1	75	0.36	commercial	DM	CM	BR	BRD
**dam line**	Broiler dam 2	75	0.35	commercial	DM	CM	BR	BRD
	Broiler dam 3	75	0.40	commercial	DM	CM	BR	BRD
	Broiler dam 4	75	0.41	commercial	DM	CM	BR	BRD
	Broiler dam 5	75	0.42	commercial	DM	CM	BR	BRD
**White**	White layer 1	75	0.24	commercial	DM	CM	LR	WL
**egg-layer**	White layer 2	75	0.27	commercial	DM	CM	LR	WL
	White layer 3	75	0.26	commercial	DM	CM	LR	WL
	White layer 4	75	0.25	commercial	DM	CM	LR	WL
	White layer 5	75	0.28	commercial	DM	CM	LR	WL
	White layer 6	75	0.21	commercial	DM	CM	LR	WL
	White layer 7	75	0.26	commercial	DM	CM	LR	WL
	White layer 8	75	0.29	commercial	DM	CM	LR	WL
	White layer 9	75	0.27	commercial	DM	CM	LR	WL
	White layer 10	75	0.22	commercial	DM	CM	LR	WL
	White layer 11	75	0.28	commercial	DM	CM	LR	WL
**Brown**	Brown layer 1	75	0.31	commercial	DM	CM	LR	BL
**egg-layer**	Brown layer 2	75	0.32	commercial	DM	CM	LR	BL
	Brown layer 3	75	0.32	commercial	DM	CM	LR	BL
	Brown layer 4	75	0.31	commercial	DM	CM	LR	BL
	Brown layer 5	75	0.37	commercial	DM	CM	LR	BL
	Brown layer 6	75	0.31	commercial	DM	CM	LR	BL
	Brown layer 7	75	0.32	commercial	DM	CM	LR	BL
	Brown layer 8	75	0.35	commercial	DM	CM	LR	BL
	Brown layer 9	75	0.32	commercial	DM	CM	LR	BL
	Brown layer 10	75	0.34	commercial	DM	CM	LR	BL
	Brown layer 11	75	0.32	commercial	DM	CM	LR	BL
**Dutch**	Groninger mew bantam	21	0.30	the Netherlands	DM	NCM	DU	DCF
	Groninger mew	22	0.28	the Netherlands	DM	NCM	DU	DCF
	Lakenvelder	46	0.27	the Netherlands	DM	NCM	DU	DCF
	Drente fowl	13	0.33	the Netherlands	DM	NCM	DU	DCF
	Assendelf fowl	22	0.28	the Netherlands	DM	NCM	DU	DCF
	Friesian fowl	9	0.33	the Netherlands	DM	NCM	DU	DCF
	Hamburgh	50	0.30	the Netherlands	DM	NCM	DU	DCF
	Polish bearded	30	0.24	the Netherlands	DM	NCM	DU	DPB
	Owl-bearded Dutch	8	0.33	the Netherlands	DM	NCM	DU	DPB
	Polish non-bearded	49	0.16	the Netherlands	DM	NCM	DU	DPB
	Breda fowl	13	0.33	the Netherlands	DM	NCM	DU	DPB
	Brabanter	50	0.34	the Netherlands	DM	NCM	DU	DPB
	Dutch bantam	23	0.32	the Netherlands	DM	NCM	DU	DPB
	Booted bantam	12	0.32	the Netherlands	DM	NCM	DU	DPB
	Barnevelder	11	0.29	the Netherlands	DM	NCM	DU	DNB
	Welsumer	41	0.31	the Netherlands	DM	NCM	DU	DNB
	North-Holland blue	34	0.33	the Netherlands	DM	NCM	DU	DNB
	Kraienkoppe	48	0.32	the Netherlands	DM	NCM	DU	DNB
	Schijndelaar	12	0.33	the Netherlands	DM	NCM	DU	DNB
**Chinese**	Bian	21	0.41	China (Inner Mongolia)	DM	NCM	CH	
	Chahua	34	0.33	China (Yunnan)	DM	NCM	CH	
	Chongren Ma	40	0.35	China (Jiangxi)	DM	NCM	CH	
	Henan Game	25	0.33	China (Henan)	DM	NCM	CH	
	Gushi	29	0.36	China (Henan)	DM	NCM	CH	
	Luyuan	30	0.38	China (Jiangsu)	DM	NCM	CH	
	Wenchang	35	0.42	China (Hainan)	DM	NCM	CH	
	Wahui	32	0.41	China (Jiangxi)	DM	NCM	CH	
	Xianju	48	0.36	China (Zhejiang)	DM	NCM	CH	
	Xiaoshan	32	0.38	China (Zhejiang)	DM	NCM	CH	

1 ) Number of individuals in genotyped DNA pool.

2 ) Average Hp based on all markers.

3 ) Name of country (province) of origin.

4 ) Breed groups for the breeds, DM = domesticated, NDM = non-domesticated, CM = commercial, NCM = non-commercial, BR = broiler, LR = layer, DU = Dutch, CH = Chinese, BRS = broiler sire line, BRD = broiler dam line, WL = white egg-layer, BL = brown egg-layer, DCF = Dutch countryfowls, DPB = Dutch polish and bearded, and DNB = Dutch new breeds.

5 ) These breeds are part of the AvianDiv project [6]. *G. g. gallus* = Aviandiv102 and *G. g. spadiceus* = Aviandiv101.

### Marker selection and allele frequency calculations

In total, 57,628 SNPs were included on the Illumina Infinium iSelect Beadchip (Table S1). For GGA1–GGA5 and GGAZ markers were selected every 20 kb; for GGA6–GGA9 every 15 kb; for GGA10–GGA14 every 11 kb; for GGA15–GGA25 every 8.5 kb; for GGA26 and GGA27 every 5 kb; and for GGA28, GGAW and the two linkage groups LGE22C19W28_E50C23 (from here on referred to as LGE22) and LGE64, every 4 kb [28]. Genotyping was performed using the standard protocol for Infinium iSelect Beadchips and raw data were analyzed with GenomeStudio v2009.2. Markers with a normalized R value of less than 0.15 were not included in further analysis. For the DNA pools, the normalized allele frequency 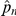 was calculated by combining the heterozygote correction equation of Hoogendoorn *et al.* [29] with the “normalization 4” equation of Peiris *et al.* [30]; 
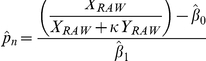
, where X_RAW_ is the raw intensity of allele A, X_RAW_ is the raw intensity of allele B, and 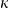 is the ratio of the average X_RAW_ and Y_RAW_ intensities based on heterozygote individuals. 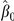 is the intercept and 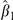 is the slope of a simple linear regression of the observed mean heterozygote-corrected frequencies based on individuals with genotype AA, AB and BB on their expected frequencies of 1, 0.5 and 0, respectively. 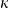, 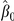 and 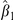 values were calculated from a panel of 458 individuals, including white and brown egg-layers, broilers, Dutch traditional breeds, *Gallus gallus spadiceus*, and *Gallus lafayetii*. If the heterozygous genotype class was missing, heterozygote correction was not performed and 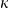 was set to 1. SNPs that were homozygous in all individual animals were removed from the data. To avoid genotype mistakes made due to technical errors, a genotype class had to contain at least three individual animals to be included in the calculation of 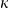, 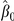 and 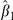. Animals from *Gallus lafayetii* were genotyped individually and genotypes were pooled *in silico* to estimate allele frequencies for this population.

### Genetic distance calculations

PHYLIP software (version 3.69 [31]) was used to calculate pairwise genetic distances between the breeds. Nei genetic distance was used as a measure for genetic distance [32]. Because PHYLIP is unable to deal with missing data, distance calculations for each pair of breeds were based on the marker data that these breeds had in common [33]. Mega 4.0 software [34] was used for hierarchical clustering using the Neighbor-Joining procedures on the genetic distance matrix for all breeds. *Gallus lafayetii* was used to root the tree.

### Signatures of selection

To decrease the influence of stochastic effects such as genetic drift, signatures of selection analysis were performed on *in silico* pooled groups of breeds. For each breed group, the allele frequency for each marker was calculated as the average for all breeds within the group. Because the allele frequencies within each breed were considered to be a good estimate of the allele frequency within the entire breed, allele frequencies for each breed within the breed groups were not weighted.

**Figure 1:**
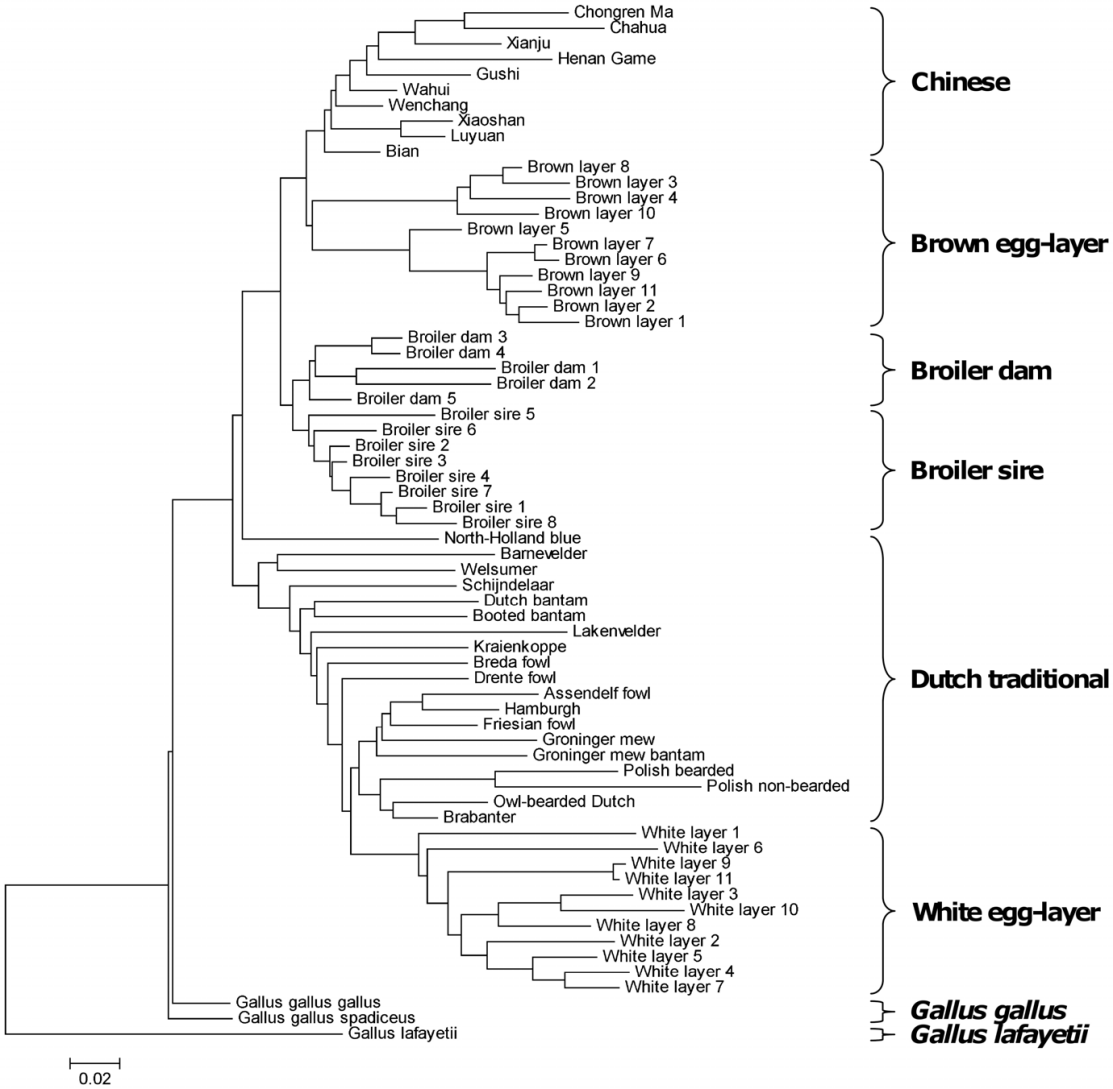
Dendrogram for the 67 breeds based on Nei genetic distance. Accolades indicate the breed groups for the clusters as used in this study.

The breeds were grouped in fourteen different breed groups at four levels (Table 1). The first level included all domesticated breeds (DM, n = 64). The two non-domesticated breeds were not grouped and analyzed because the group size was too small. The second level was based on their commercial background and included commercial (CM, n = 35) and non-commercial (NCM, n = 29) breeds. The third level was based on either their general commercial purpose or geographical location and included broiler (BR, n = 13), layer (LR, n = 22), Dutch traditional (DU, n = 19) and Chinese (CH, n = 10) breeds. The fourth level was based on either their position in the dendrogram (Figure 1) and included the broiler sire lines (BRS, n = 8), broiler dam lines (BRD, n = 5), white egg-layers (WL, n = 11) and brown egg-layers (BL, n = 11), or were based on their classical classification and included the Dutch countryfowls (DCF, n = 8), Dutch polish and bearded (DPB, n = 5), and Dutch new breeds (DNB, n = 6). Because effective population sizes for the breeds are unknown or highly uncertain, we did not account for total breed size in our analysis, resulting in equal contributions per breed to the breed group.

To identify regions under selection the “Z transformed heterozygosity” (ZHp) approach was used as previously described [1]. Briefly, in an overlapping sliding window approach (overlap to the consecutive window is the number of markers per window - 1) the heterozygosity 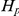 was calculated as: 
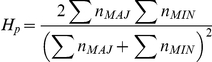
, where 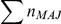 is the sum of major allele frequencies, and 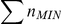 is the sum of the minor allele frequency within a window. Individual 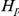 values were Z-transformed: 
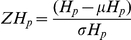
, where 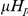 is the overall average heterozygosity and 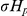 is the standard deviation for all windows within one breed group. We applied a window size of 5, 10, 20, 50 and 100 markers to identify regions under putative selection. The ZHp threshold values for suggestive (ZHp less or equal to -4) and strong (ZHp less or equal to -6) evidence were used because they represent the extreme lower end of the distribution (Figure S1).

## Results

From the 57,628 SNPs that were included on the chip, 51,076 were used for the selective sweep analysis. Because the breed pools included both female and male individuals, the analyses were only performed on autosomal markers and therefore the 3,023 markers located on chromosome W and Z were excluded from the analysis. Moreover, 1,136 markers were unmapped on the current genome build and were excluded from the analysis. A total of 2,389 markers were excluded as they were either homozygous in all individual animals (n = 2,146) or did not pass the quality control (n = 243). Linkage group LGE64 consisted of only 4 markers and was not included in further analysis.

The 51,076 autosomal SNPs were used to construct a tree representing genetic distances between 67 breeds (Figure 1). The two RJF subspecies and *Gallus lafayetii* cluster separate from the domesticated breeds. The domesticated breeds are divided in two branches. Brown egg-layers, broilers and Chinese breeds cluster together in one branch while white-egg layers and Dutch traditional breeds cluster in the other. Within the broiler cluster, a clear distinction was found between the broiler sire and broiler dam lines highlighting that they were derived from different breeds. The Dutch traditional breeds cluster together according to their classical classification [35] with a few exceptions.

**Figure 2:**
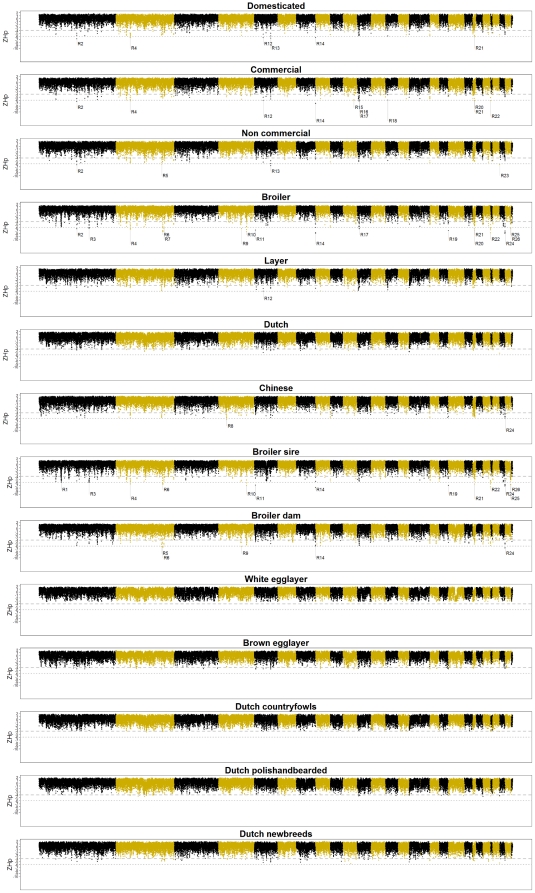
ZHp values for each breed group using a sliding window of five markers across the genome. Odd chromosomes numbers (and LGE22) are shown in red and even chromosome numbers are shown in blue. The grey dotted line indicates a ZHp threshold value of -4 or -6. For the regions with strong evidence of selection the ID is shown beneath the plot.

To identify regions that are likely to be or have been under selection, Hp and ZHp values were calculated for a number of different marker window sizes for all fourteen breed groups (Table S2, S3, S4, S5, S6, S7, S8, S9, S10, S11). Based on these analyses we decided to primarily focus on a size of five markers per window (Figure 2), unless chromosomal regions with strong evidence of selection were not identified in a particular breed group (see below). With a window size of five markers, the distribution of the Hp values resembled a normal distribution for most breed groups (Figure S2) while providing a high resolution to detect potential candidate genes that might have been under selection. A marker window size of five also enabled us to identify the proven selective sweep at the *BCDO2* locus with strong evidence in the commercial breed group [12]. Increasing the window size resulted in the loss of identification of this locus within the commercial breed group (Table S12). Average sizes for the five marker windows were; 97 kb for GGA1–5, 71 kb for GGA6–10, 46 kb for GGA11–20, and 31 kb for GGA21-GGA28 and linkage group LGE22. These sizes are comparable to the average length (∼60 kb) of previously identified selective sweeps in the chicken genome [1]. For a window size of five markers, 396 regions were identified after merging consecutive (sliding) windows where at least one breed group showed suggestive evidence of selection (Table S12). In total, 26 regions showed strong evidence of selection (Table 2, Table S12). Three of these regions (R11, R25, and R26) were found exclusively within the broiler breed groups. All three showed strong evidence of selection in the broiler sire line and R11 also showed weak evidence of selection in the broiler dam line. Region R1 showed strong evidence for selection exclusively within the broiler sire breed group. Region R8 showed strong evidence of selection exclusively within the Chinese breed group. The average overall heterozygosity (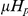) and standard deviation (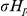) for the fourteen breed groups are shown in Table S13. The average heterozygosity for each breed was based on all markers and is shown in Table 1.

**Table 2:** Regions of putative selection identified in this study using a window size of 5 markers.

ID	Chromosome	Position (Mb)^1^	DM	CM	NCM	BR	LR	DU	CH	BRS	BRD	WL	BL	DCF	DPB	DNB
R1	1	57.16–57.64	−3.5	−3.2	−3.3	**−5.8**	−1.2	−3.8	−2.7	**−6.1**	−3.7	−0.9	−2.2	−2.6	−3.7	−3.3
R2	1	98.80–98.95	**−6.8**	**−6.5**	**−6.3**	**−6.5**	−4.7	−4.4	−5.4	−5.6	−5.8	−1.7	−4.5	−3.7	−4.1	−3.6
R3	1	131.15–131.59	−3.6	−4.1	−2.9	**−6.3**	−2.1	−3.5	−2.9	**−6.1**	−5.2	−3.1	−2.1	−3.7	−2.0	−4.0
R4	2	35.22–35.69	**−6.5**	**−6.3**	−5.7	**−6.6**	−4.7	−3.7	−5.8	**−6.6**	−4.8	−2.8	−3.9	−3.2	−4.3	−3.8
R5	2	120.56–120.87	−5.6	−4.9	**−6.1**	−2.0	−5.9	−4.0	−5.7	−0.7	**−6.2**	−2.9	−4.5	−3.2	−2.3	−4.9
R6	2	123.46–123.90	−5.3	−5.3	−4.6	**−7.4**	−3.1	−4.6	−2.2	**−6.7**	**−6.5**	−3.1	−1.1	−4.2	−3.4	−4.3
R7	2	126.69–126.91	−4.6	−4.2	−4.4	**−6.4**	−2.1	−3.0	−3.7	−5.6	−5.6	−0.6	−3.3	−1.9	−2.2	−4.3
R8	4	22.27–22.47	−2.0	−1.4	−2.5	−2.3	−0.5	−1.1	**−6.3**	−2.0	−1.7	0.0	−1.8	−0.5	−1.9	0.0
R9	4	60.47–60.60	−5.7	−5.7	−4.6	**−6.4**	−3.9	−2.9	−4.0	−5.3	**−6.1**	−1.5	−3.2	−2.9	−1.7	−4.1
R10	4	73.63–73.88	−0.8	0.0	−1.6	**−7.4**	0.7	−0.8	−4.9	**−7.1**	−5.6	−0.5	0.0	−1.6	−1.0	−1.3
R11	5	2.34–2.51	−0.5	−2.0	1.0	**−6.9**	0.2	1.3	−2.9	**−7.0**	−4.6	0.4	0.9	1.2	1.3	0.1
R12	5	24.50–24.71	**−6.5**	**−6.1**	−5.4	−3.7	**−6.0**	−5.2	−2.2	−3.1	−3.4	−3.2	−4.5	−4.2	−4.1	−5.4
R13	5	44.05–44.33	**−6.6**	−5.7	**−6.7**	−3.9	−5.0	−4.5	−5.7	−2.2	−5.2	−1.9	−4.6	−3.4	−3.5	−5.2
R14	7	38.03–38.35	**−6.4**	−**7.2**	−4.4	**−7.9**	−5.0	−3.8	−2.9	**−7.8**	**−6.0**	−3.1	−3.4	−2.5	−4.0	−3.3
R15	10	17.52–17.59	−4.5	**−6.1**	−3.0	−4.0	−5.7	−2.6	−2.1	−3.1	−3.8	−2.9	−4.5	−1.7	−2.3	−2.4
R16	11	2.53–2.67	−5.7	**−6.1**	−4.3	−5.8	−4.7	−3.0	−3.4	−5.3	−4.6	−2.1	−3.9	−2.6	−2.0	−3.6
R17	11	3.37–3.55	−5.6	**−6.4**	−3.7	**−6.5**	−4.7	−3.1	−1.6	−5.6	−5.9	−2.4	−3.5	−2.6	−1.8	−3.9
R18	13	3.82–3.93	−5.9	**−6.0**	−4.9	−4.0	−5.6	−3.2	−4.2	−3.5	−3.4	−3.1	−3.8	−2.5	−2.1	−3.5
R19	19	9.81–9.92	−2.8	−4.5	−1.1	**−7.0**	−2.2	0.6	−5.3	**−7.1**	−4.8	0.6	−4.5	0.4	0.1	−1.7
R20	22	1.95–2.01	−5.7	**−6.3**	−3.8	**−6.3**	−4.7	−1.7	−5.0	−5.6	−5.4	−2.4	−3.5	−1.0	−1.0	−2.4
R21	22	2.15–2.22	**−6.2**	**−6.1**	−5.0	**−6.9**	−4.2	−3.6	−4.1	**−6.6**	−5.3	−2.6	−2.3	−2.6	−2.5	−4.8
R22	24	6.25–6.31	−1.6	**−6.2**	0.3	**−7.8**	−3.9	0.5	−2.0	**−7.5**	−5.9	−0.9	−4.2	−1.1	−2.2	−2.6
R23	26	5.01–5.09	−5.5	−4.2	**−6.3**	−3.8	−3.1	−5.4	−3.2	−2.9	−3.9	−0.9	−2.7	−4.1	−4.5	−5.6
R24	27	4.61–4.84	−5.3	−4.9	−5.0	**−8.0**	−3.2	−3.1	**−6.0**	**−7.3**	**−6.7**	−2.3	−4.3	−2.3	−2.0	−4.5
R25	28	3.75–3.80	−1.0	−1.3	−1.8	**−6.2**	−0.2	−1.5	−1.6	**−6.7**	−3.8	−1.4	−0.1	−1.5	−1.4	−1.2
R26	28	4.04–4.07	−2.6	−2.2	−2.6	**−6.2**	0.0	−1.3	−3.4	**−6.7**	−3.6	1.1	−1.0	−0.8	−0.2	−3.6

1 ) Position based on chicken genome build WASHUC2. ZHp values are shown for each region for each breed group. Values in bold values are less than or equal to -6. DM = domesticated, CM = commercial, NCM = non-commercial, BR = broiler, LR = layer, DU = Dutch, CH = Chinese, BRS = broiler sire line, BRD = broiler dam line, WL = white egg-layer, BL = brown egg-layer, DCF = Dutch countryfowls, DPB = Dutch polish and bearded, and DNB = Dutch new breeds.

With a window size of five markers, chromosomal regions with strong evidence of selection were not identified within the DU, DCF, DPB, DNB, WL, or BL breed groups. For the DCF, DPB, WL, and BL breed groups, these regions were not identified even if the window size was increased to 100 markers (Table S6). Four regions in the DU and DNB breed groups were identified (if regions within a breed group overlapped between the different marker window sizes, the smallest region was considered to be the putative region under selection) that showed strong evidence for selection if the window size was increased to 10 or 20 markers (Table S12). For the DU breed group, a 558 kb region was identified on chromosome 15 (ZHp = −6.2, window size = 20). For the DNB breed group, a region of 775 kb was identified on chromosome 1 (ZHp = −6.4, window size = 10), a region of 538 kb was identified on chromosome 5 (ZHp = −6.2, window size = 10), and a region of 1084 kb was identified on chromosome 22 (ZHp = −6.2, window size = 20). The four regions identified in the DU and DNB breed groups were all overlapping regions of suggestive significance identified within the analysis based on a five marker window.

## Discussion

The position of the breeds in the dendrogram (Figure 1) is largely in agreement with previous published data [36] and the expected historical origin of the breeds. The broilers and brown egg-layers cluster between the Chinese breeds on one side and the Dutch and white egg-layers on the other side. The broiler and brown egg-layer breeds were established in the late 19^th^ and early 20^th^ century by crossing European breeds with Asian breeds [13], [16] which was confirmed by molecular evidence [27]. The Dutch traditional and white egg-layer breeds both have their origin in Europe [13], [16], although some East- and Southeast Asian influences have been found in a few breeds [27]. We used an *in silico* pooling approach of populations, defining groups based on overall genetic relatedness to decrease stochastic effects, such as genetic drift, in our analysis. If a region under selection is present in only one breed, it will be averaged out due to a high diversity in the other breeds included in the same breed group. However, if a region is present in all breeds, the confidence that this region is truly under selection will increase.

Although we identified regions of strong selection within most breed groups (window size of five markers) the Dutch (either separate or in the classification breed groups) and white- and brown egg-layer breed groups did not have these regions. The Dutch breeds have been bred using a variety of breeding goals making it difficult to identify regions with strong evidence of selection. These differing breeding goals may cause there to be little overlap in selected regions between the breeds within the breed group. The lack of identification of regions under selection within the white and brown egg-layers breed groups most likely results from the origin of the breeds. Both the white and brown egg-layers breeds were created using a small base population and this founder effect resulted in a major population bottleneck [16]. Regions with low genetic diversity caused by the bottleneck will exist in all breeds derived from the base population. Our method relies on the heterozygosity of a given marker window being an outlier compared to the average heterozygosity of the genome. Many low diversity regions will lower the average heterozygosity and increase the standard deviation making the identification of outlier genomic regions more challenging. In our analysis the standard deviation (based on the heterozygosity of all bins) decreased with an increasing number of markers per window (Table S13), potentially enhancing the identification of outlier genomic regions. Indeed, for DU and DNB breed groups, an increased window size resulted in the identification of four chromosomal regions with strong evidence of selection (Table S12). However, the regions identified with a window size of 20 markers included many genes, making it difficult to comment on possible candidates. The two regions identified with a window size of 10 did not include interesting functional candidate genes. Chromosomal regions with strong evidence of selection were not identified in the DCF, DPB, WL, and BL, even if the window size was increased to 100 markers (Table S12). Thus, independent of the window size applied, it remains challenging to identify regions with strong evidence of selection in breeds that either experienced a common population bottleneck or that lack common breeding goals. To identify regions under selection in the egg-layers, we combined the two breed groups of the white and brown egg-layers. Both egg-layers have been selected for similar production traits related to egg production and combining these two breeds groups could lead to the identification of genomic regions independently being selected that result in similar egg production traits. Indeed, the number of regions showing suggestive (11 over 8) and strong (1 over 0) evidence of selection was increased by this strategy.

While Broiler and Layer populations were homogenous regarding the number of individuals within each DNA pool, the sample sizes of Dutch and Chinese populations were not. For breed group including Dutch and Chinese breeds we investigated the effects of unweighted vs. weighted pooling strategies (weight on the number of individuals within each population pool). The correlations between the unweighted and weighted strategies were very high (the correlation coefficient (Pearson) for the ZHp based on a five marker windows were 0.996, 0.980, 0.966, 0.953, and 0.942 for the CH, DU, DCF,DPB and DNB breed pools, respectively), thereby confirming the validity of the unweighted pooling strategy for all breed groups.

Of the 26 regions that show strong evidence of selection in the breed groups (window size of five markers, Table S12), 13 were previously described [1]. The identification of these regions using various detection methods implies that these regions have indeed undergone a selective sweep. Some of these regions contain genes with biological functions that were previously linked to traits under selection in the chicken. For example, *IGF1* [37], [38] and *PMCH* [39] detected within region R1, and *BCDO2* [12] detected within R22. Other genes that are located within putative regions under selection are not specifically described in literature but nevertheless have biological functions that can be linked to production traits in chicken. *HNF4G* (region R6) knockout mice have a higher bodyweight at 7 weeks and a reduced feed and water intake compared to wild type mice [40]. Additionally, a quantitative trait locus (QTL) associated with bodyweight from 3 to 7 weeks overlaps region R6 [41]. Given the biological function of *HNF4G*, the putative selective sweep detected at R6 might have been a direct consequence of selection for bodyweight traits. Region R11 is embedded within the gene encoding NEL-like 1 (*NELL1*). *NELL1* is involved in bone tissue formation and *NELL1*-deficient mice have skeletal defects in the cranial vault, vertebral column and ribcage [42], [43]. The biological functions of *NELL1* may relate to selection on the skeletal integrity of modern broilers. Skeletal integrity is likely to have been co-selected with growth rate and meat yield as the skeleton of modern broilers needs to support more weight [44]. Animals not capable of dealing with the increasing bodyweight are likely to develop defects such as tibial dyschondroplasia, valgus-varus deformity and spondylolisthesis [20] and will be rejected from the breeding program. This rejection will essentially lead to a positive selection for skeletal integrity. Heavy birds are more prone to develop leg problems, and it is therefore expected that selection was strongest in heavier breeds. This is in agreement with region R11 which shows strong evidence of selection in the heavy broiler sire lines and weak evidence in the slightly lighter broiler dam lines. QTLs that are associated with bone or skeletal traits have not been detected near region R11 [45].

Besides the 13 regions that were previously described, 13 additional regions with strong evidence of selection were identified in this study. Conversely, several regions with strong evidence of selection found previously [1] were not identified in our study. These differences in identified regions may be due to the different methods used. While our study was based on genotyping many breeds with a SNP genotyping assay, the study of Rubin et al. [1] was based on low coverage whole-genome re-sequencing of a small number of breeds. Regions identified in our study might be poorly covered in the massively parallel sequencing strategy or might have not been detected because the breeds were not included. In addition, we included more breeds per breed group which might reduce the false positive regions found as a result of genetic drift.

While the approach described in this study has several strong advantages – the ability to include many different populations cost-effectively being among the most important – the application of SNP based assays has limitations, notably ascertainment bias and low marker resolution. The SNPs genotyped for our analysis were discovered in two independent studies. One study compared the *G. gallus* genome sequence derived from a single RJF to one Silkie, one white egg-layer, or one broiler [46]. The second study sequenced four pools of commercial chicken using massively parallel sequencing (two broiler lines, a white egg layer line, and a brown egg layer line (MAMG, unpublished). In both studies a SNP was identified when a single nucleotide polymorphism was observed between the reference RJF and one of the four discovery breeds. This ascertainment process inevitably results in SNPs having a higher likelihood of being polymorphic in the genotyping assay in some breeds over others. The selection of markers that are eventually included in the genotyping assay also introduces a bias. Selection criteria for the SNPs are mainly based on their minor allele frequency (MAF) and position on the genome. SNPs that are near fixation in the four SNP discovery breeds will have a low MAF, as well as when they are nearly fixed for the non-reference allele. Because all four SNP discovery breeds represent domesticated breeds, particularly regions under selection due to domestication will be underrepresented since SNPs within these regions will have low MAF and are not included in the genotyping assay.

Although markers are selected evenly throughout the genome, the resolution of the assay will be insufficient to identify all regions of selection. The genomic size of selective sweeps is positively correlated to selection pressure and negatively with recombination rate. Genomic regions under strong and recent directional selection located in relatively poorly recombining regions of the genome (e.g., the macro-chromosomes in birds compared to the micro-chromosomes [26], [47], [48]) will be detected much more easily). Although the *TSHR* selective sweep was previously found to have resulted in 40 kb without polymorphisms in most domestic breeds [1], we were unable to identify this locus. In our analysis only one SNP is within this 40 kb region and although this SNP is fixed in almost all domesticated breeds, the window(s) that included this SNP never reached significance as the other markers in the window are segregating at relatively high frequencies. Although the massively parallel sequencing strategy does not suffer from the ascertainment bias described above, the high costs of this method currently restrict the number of breeds that can be included in the analysis. In this study, we specifically chose the less expensive SNP assays in order to increase the total number of breeds. Not only were we able to comment on a wide variety of breeds, the increased number of breeds within a breed group enabled us to decrease the influence of stochastic effects such as genetic drift.

In our data we identified five regions (R1, R8, R11, R25, and R26) that are specific for one breed group (Table 2, Figure 2). Because these regions are not subjected to the possible bias of breed specific markers, we consider these to be reliable. Otherwise there would be signatures of selection in all but one breed group. Two regions (R1 and R11) have already been discussed above. Region R8 shows strong evidence of selection and is specific for the Chinese breeds and includes the gene encoding platelet derived growth factor C (*PDGFC*), Platelet derived growth factors are major mitogens and stimulants of motility in mesenchymal cells [49], [50], cells that can differentiate into a variety of cell types including bone and fat cells. In mice, *PDGFC* is widely expressed in mesenchymal precursors and the myoblast of the smooth and skeletal muscles [51]. Near region R8 several QTLs are detected in chicken (although not within Chinese breeds) [45]. Among these QTL are shank length [52], tibia strength [52], and thigh muscle weight [53], [54]. Given the biological function of *PDGFC*, we propose this gene as a candidate gene for follow-up studies for these traits. Due to the broad range of biological functions of the genes located within R25 and R26, we do not propose candidate genes that might have been under selection because of their biological function.

In conclusion, based on a window size of five makers, we identified 396 regions of putative selection within the chicken genome and 26 of these regions show strong evidence of selection in at least one of the fourteen breed groups. Our approach demonstrates the strength of including many different populations with similar, and breed groups with different selection histories to reduce stochastic effects based on single populations. The identification of the regions of putative selection detected several candidate genes that could aid in further improvement of production traits and disease resistance.

## Supporting Information

Figure S1 **Distribution of ZHp values for all windows sizes.**
(PDF)

Figure S2 **Distribution of Hp values for all windows sizes.**
(PDF)

Table S1 **Information on all SNP markers used.** Chromosomal locations are based on the position in the WASHUC2 build.
(XLS)

Table S2 **ZHp values for all windows containing 5 markers.** Chromosomal locations are based on the position in the WASHUC2 build. DM = domesticated, CM = commercial, NCM = non-commercial, BR = broiler, LR = layer, DU = Dutch, CH = Chinese, BRS = broiler sire line, BRD = broiler dam line, WL = white egg-layer, BL = brown egg-layer, DCF = Dutch countryfowls, DPB = Dutch polish and bearded, and DNB = Dutch new breeds.
(TXT)

Table S3 **ZHp values for all windows containing 10 markers.** Chromosomal locations are based on the position in the WASHUC2 build. DM = domesticated, CM = commercial, NCM = non-commercial, BR = broiler, LR = layer, DU = Dutch, CH = Chinese, BRS = broiler sire line, BRD = broiler dam line, WL = white egg-layer, BL = brown egg-layer, DCF = Dutch countryfowls, DPB = Dutch polish and bearded, and DNB = Dutch new breeds.
(TXT)

Table S4 **ZHp values for all windows containing 20 markers.** Chromosomal locations are based on the position in the WASHUC2 build. DM = domesticated, CM = commercial, NCM = non-commercial, BR = broiler, LR = layer, DU = Dutch, CH = Chinese, BRS = broiler sire line, BRD = broiler dam line, WL = white egg-layer, BL = brown egg-layer, DCF = Dutch countryfowls, DPB = Dutch polish and bearded, and DNB = Dutch new breeds.
(TXT)

Table S5 **ZHp values for all windows containing 50 markers.** Chromosomal locations are based on the position in the WASHUC2 build. DM = domesticated, CM = commercial, NCM = non-commercial, BR = broiler, LR = layer, DU = Dutch, CH = Chinese, BRS = broiler sire line, BRD = broiler dam line, WL = white egg-layer, BL = brown egg-layer, DCF = Dutch countryfowls, DPB = Dutch polish and bearded, and DNB = Dutch new breeds.
(TXT)

Table S6 **ZHp values for all windows containing 100 markers.** Chromosomal locations are based on the position in the WASHUC2 build. DM = domesticated, CM = commercial, NCM = non-commercial, BR = broiler, LR = layer, DU = Dutch, CH = Chinese, BRS = broiler sire line, BRD = broiler dam line, WL = white egg-layer, BL = brown egg-layer, DCF = Dutch countryfowls, DPB = Dutch polish and bearded, and DNB = Dutch new breeds.
(TXT)

Table S7 **Hp values for all windows containing 5 markers.** Chromosomal locations are based on the position in the WASHUC2 build. DM = domesticated, CM = commercial, NCM = non-commercial, BR = broiler, LR = layer, DU = Dutch, CH = Chinese, BRS = broiler sire line, BRD = broiler dam line, WL = white egg-layer, BL = brown egg-layer, DCF = Dutch countryfowls, DPB = Dutch polish and bearded, and DNB = Dutch new breeds.
(TXT)

Table S8 **Hp values for all windows containing 10 markers.** Chromosomal locations are based on the position in the WASHUC2 build. DM = domesticated, CM = commercial, NCM = non-commercial, BR = broiler, LR = layer, DU = Dutch, CH = Chinese, BRS = broiler sire line, BRD = broiler dam line, WL = white egg-layer, BL = brown egg-layer, DCF = Dutch countryfowls, DPB = Dutch polish and bearded, and DNB = Dutch new breeds.
(TXT)

Table S9 **Hp values for all windows containing 20 markers.** Chromosomal locations are based on the position in the WASHUC2 build. DM = domesticated, CM = commercial, NCM = non-commercial, BR = broiler, LR = layer, DU = Dutch, CH = Chinese, BRS = broiler sire line, BRD = broiler dam line, WL = white egg-layer, BL = brown egg-layer, DCF = Dutch countryfowls, DPB = Dutch polish and bearded, and DNB = Dutch new breeds.
(TXT)

Table S10 **Hp values for all windows containing 50 markers.** Chromosomal locations are based on the position in the WASHUC2 build. DM = domesticated, CM = commercial, NCM = non-commercial, BR = broiler, LR = layer, DU = Dutch, CH = Chinese, BRS = broiler sire line, BRD = broiler dam line, WL = white egg-layer, BL = brown egg-layer, DCF = Dutch countryfowls, DPB = Dutch polish and bearded, and DNB = Dutch new breeds.
(TXT)

Table S11 **Hp values for all windows containing 100 markers.** Chromosomal locations are based on the position in the WASHUC2 build. DM = domesticated, CM = commercial, NCM = non-commercial, BR = broiler, LR = layer, DU = Dutch, CH = Chinese, BRS = broiler sire line, BRD = broiler dam line, WL = white egg-layer, BL = brown egg-layer, DCF = Dutch countryfowls, DPB = Dutch polish and bearded, and DNB = Dutch new breeds.
(TXT)

Table S12 **All regions of putative selection found for the different window sizes.** Chromosomal locations are based on the position in the WASHUC2 build. Size refers to the total size of the merged windows. # windows refer to the number of merged windows. Region ID refers to the region with strong evidence of selection as described in this manuscript (window size 5 only). DM = domesticated, CM = commercial, NCM = non-commercial, BR = broiler, LR = layer, DU = Dutch, CH = Chinese, BRS = broiler sire line, BRD = broiler dam line, WL = white egg-layer, BL = brown egg-layer, DCF = Dutch countryfowls, DPB = Dutch polish and bearded, and DNB = Dutch new breeds. Values of Rubin et al. refers to ZHP values found in a previous study [1] (window size 5 only). The ‘genes’ column include information of the genes included in the region of putative selection (window size 5 only). For each gene the location within the region is given followed by the Ensembl chicken ID and human orthologs name if known. (1) gene is located within region, (2) region is located within gene, (3) region overlaps 5′ end of gene, and (4) region overlaps 3′end of gene. *) human 1:many orthologs **) human many:many orthologs.
(XLS)

Table S13 **The average overall heterozygosity and standard deviation for all fourteen breed groups for all different window sizes.**
(XLS)
