# Supplementary material for: Signatures of Selection in the Genomes of Commercial and Non-Commercial Chicken Breeds
Source: PLoS One. 2012 Feb 27;7(2):e32720. doi: 10.1371/journal.pone.0032720 (PMC3287981; doi:10.1371/journal.pone.0032720)

Hp distribution window of 5 markers

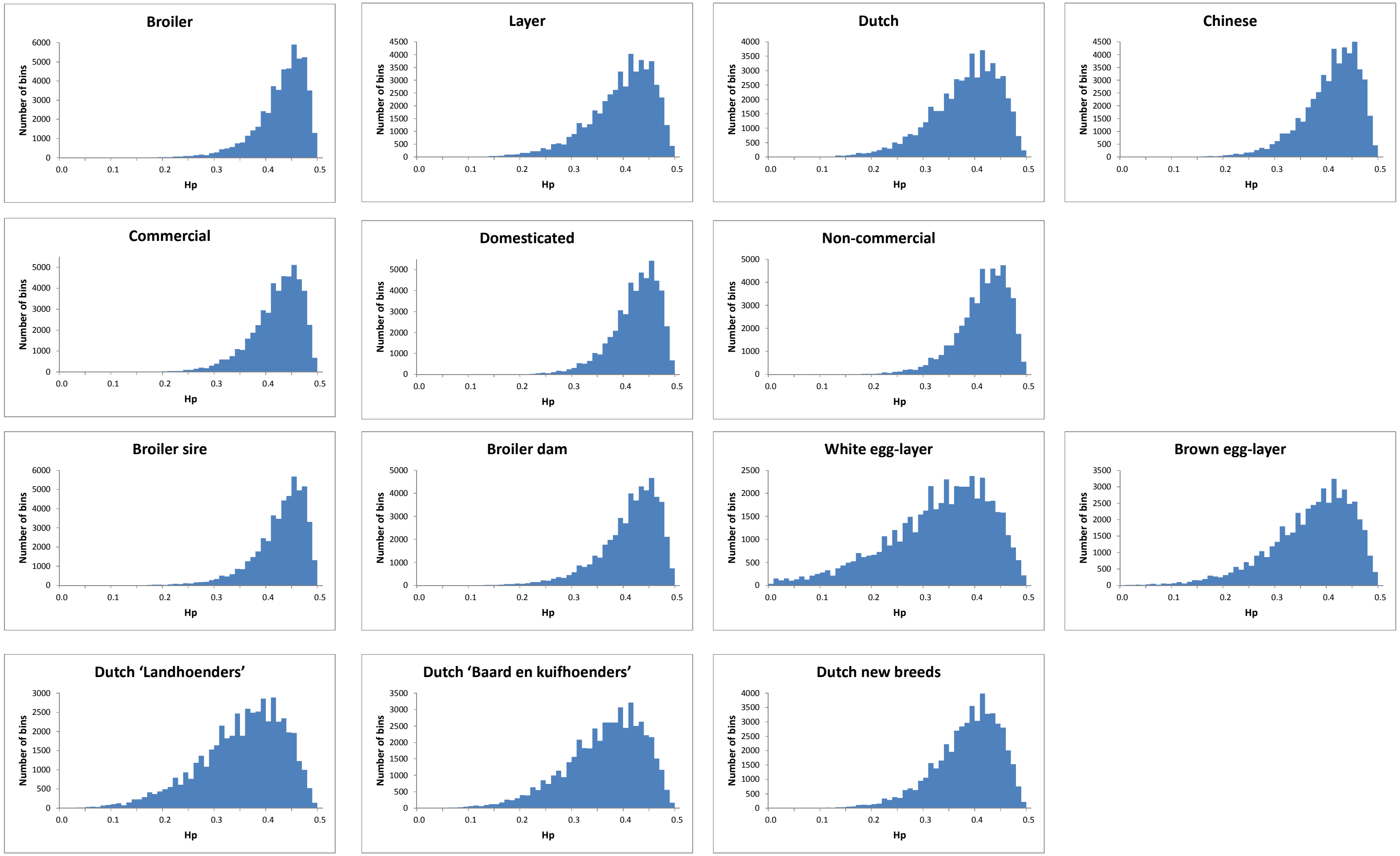

Hp distribution window of 10 markers

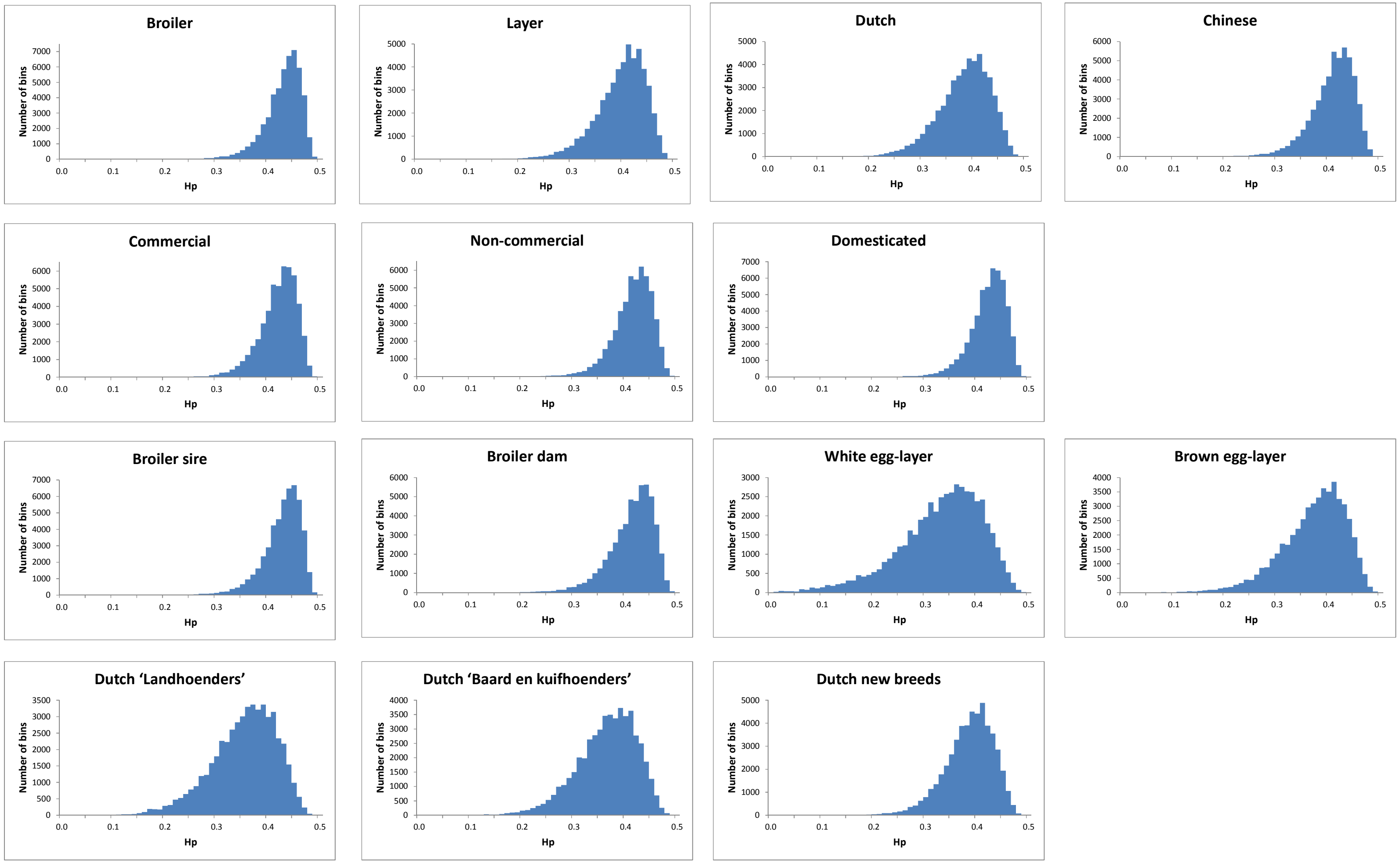

Hp distribution window of 20 markers

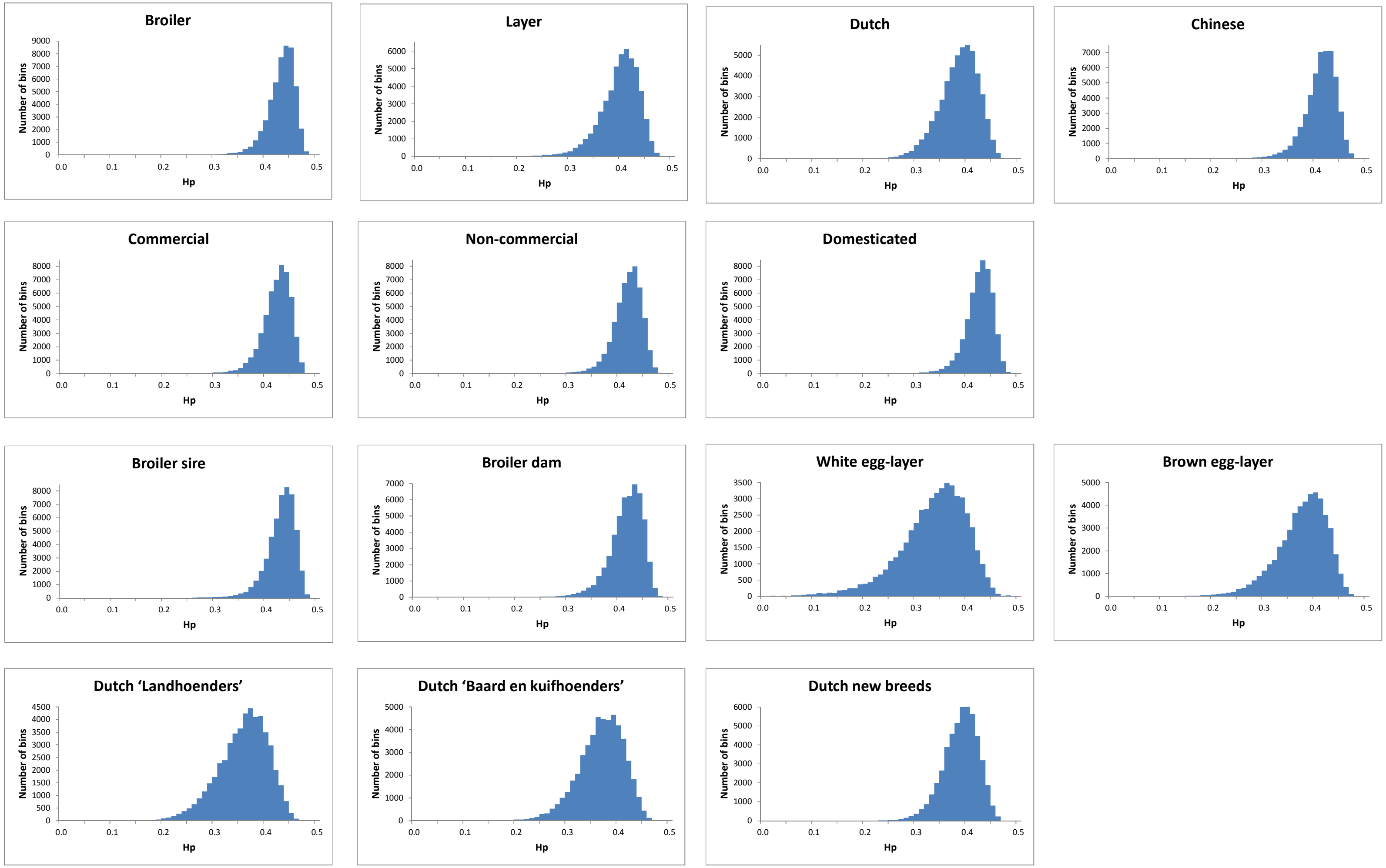

Hp distribution window of 50 markers

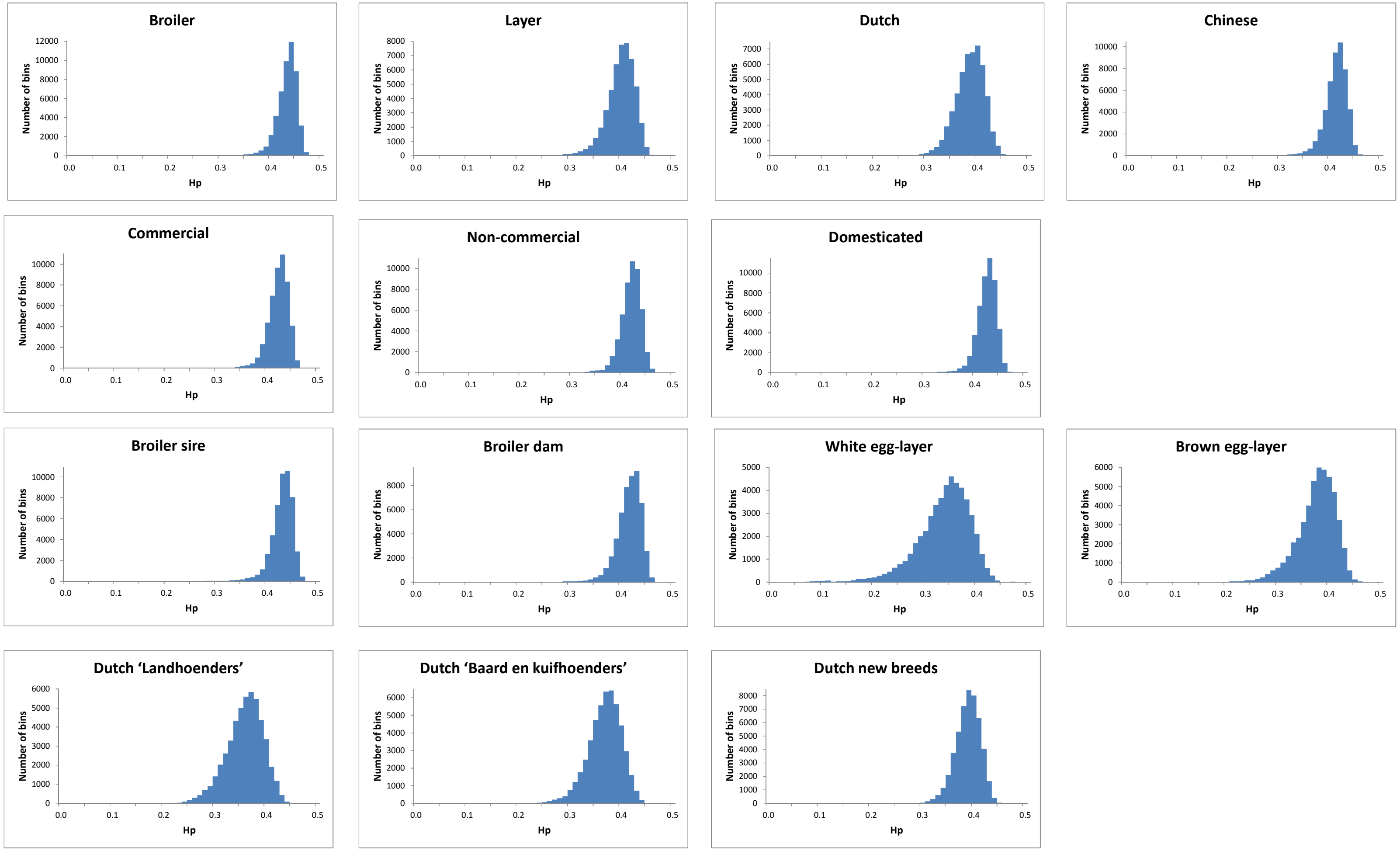

Hp distribution window of 100 markers

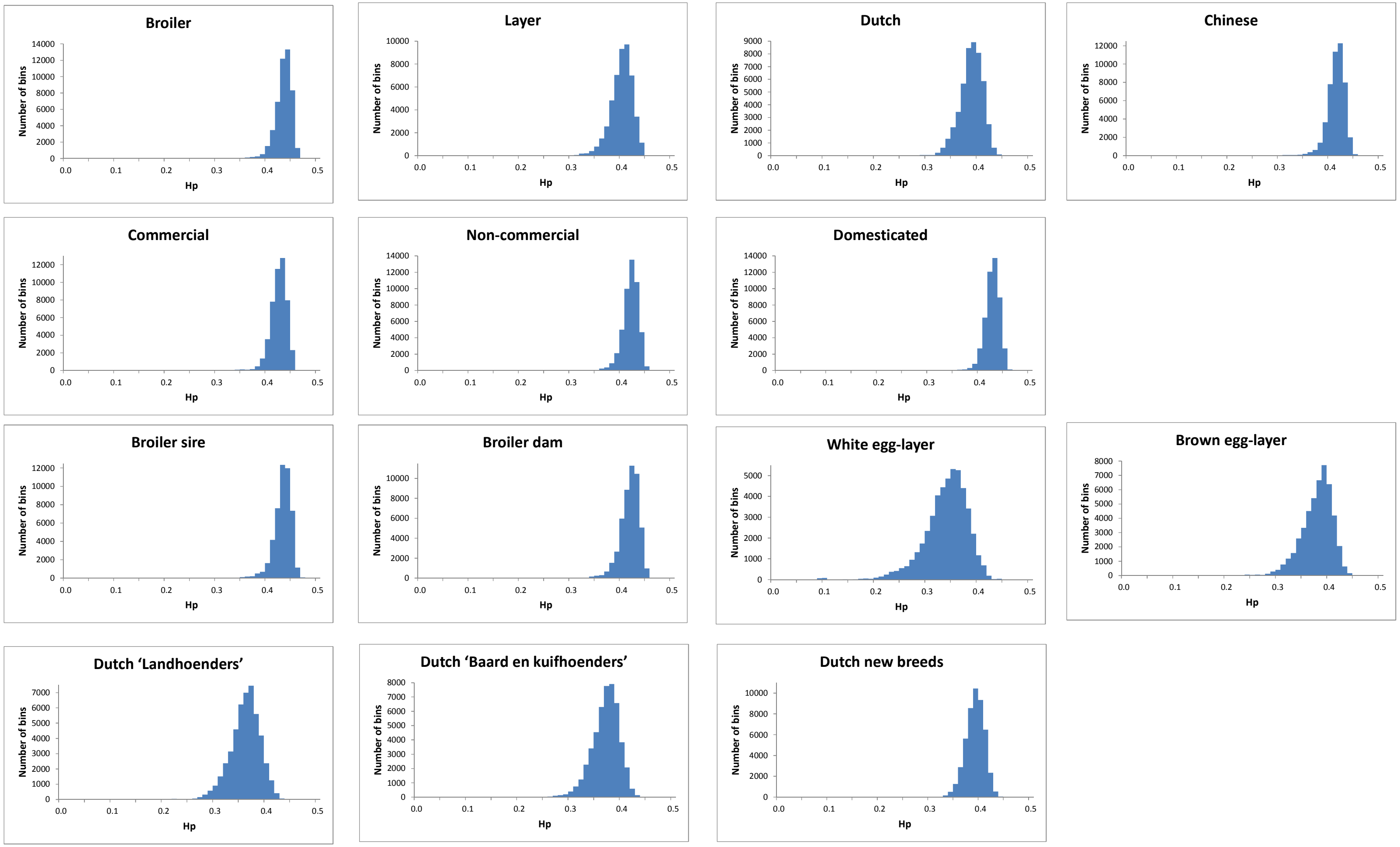

Supplement: Figure S2 — Distribution of Hp values for all windows sizes. (PDF) [file pone.0032720.s002.pdf]
